# GLP‐1, GIP, and Glucagon Excursions During a Mixed Meal Tolerance Test in Young and Lean South Asians Versus Europids

**DOI:** 10.1111/dom.70704

**Published:** 2026-04-15

**Authors:** Carlijn A. Hoekx, Lisa B. D. Brinkman, Robin van Eenige, Sander Kooijman, Marcel H. A. Muskiet, Ingrid M. Jazet, Borja Martinez‐Tellez, Patrick C. N. Rensen, Mariëtte R. Boon

**Affiliations:** ^1^ Division of Endocrinology, Department of Medicine Leiden University Medical Center Leiden the Netherlands; ^2^ Einthoven Laboratory for Experimental Vascular Medicine Leiden University Medical Center Leiden the Netherlands; ^3^ Department of Nursing Physiotherapy and Medicine SPORT Research Group (CTS‐1024), CIBIS Research Center, University of Almería Almería Spain; ^4^ Biomedical Research Unit Torrecárdenas University Hospital Almería Spain; ^5^ CIBER de Fisiopatología de la Obesidad y Nutrición (CIBEROBN), Instituto de Salud Carlos III Granada Spain; ^6^ Obesity Center CGG Erasmus MC Rotterdam the Netherlands; ^7^ Division of Endocrinology, Department of Internal Medicine Erasmus MC Rotterdam the Netherlands

**Keywords:** energy metabolism, incretin hormones, mixed meal test, South Asian

## Abstract

**Aims:**

South Asians exhibit an unfavourable metabolic phenotype characterized by visceral obesity, insulin resistance and dyslipidemia. While various hormones play a critical role in regulating postprandial energy metabolism, it remains unclear whether they respond differently to food intake. We aimed to compare the meal‐induced excursion of incretin hormones (GLP‐1 and GIP) and glucagon between South Asians and Europids.

**Materials and Methods:**

Forty nine young, lean South Asian (*n* = 24), and Europid (*n* = 25) males and females underwent an extended (up to 240 min) mixed meal tolerance test (MMTT). At seven time points circulating incretins (active and total GLP‐1 and GIP), glucagon, and parameters related to glucose (i.e., glucose, insulin) and lipid metabolism were measured.

**Results:**

In response to the MMTT, Europids generally exhibited a single peak in glucose levels at t = 30 min, while South Asians tended to display a biphasic glucose response, with peaks at t = 30 and t = 90 min. Among South Asian males, this was accompanied by an increased insulin response, characterized by elevated levels at the corresponding glucose peaks. South Asian females, however, demonstrated a marked drop in circulating glucagon at t = 90 min, and biphasic excursions of total and active GLP‐1 and GIP (t = 30 and t = 120 min). Postprandial lipid excursions did not differ between ethnicities.

**Conclusions:**

In contrast to a monophasic glucose response to the MMTT of Europids, South Asians tended to exhibit a biphasic glucose response, with sex‐specific hormonal patterns, suggesting altered incretin and insulin dynamics despite similar postprandial lipid excursions.

**Trial Registration:**

ClinicalTrials.gov (NCT05829018; registration date: 25‐04‐2023)

## 1 Introduction

Currently, ~16% of adults worldwide live with obesity [1], a prevalence that continues to rise [1, 2]. Obesity not only impacts health directly but also increases the risk of various obesity‐related diseases, such as type 2 diabetes mellitus (T2DM) and cardiovascular diseases [3, 4].

The South Asian population is disproportionately affected by obesity and obesity‐related diseases. Epidemiological studies indicate that South Asians have a fourfold higher risk of developing T2DM compared to other ethnicities [5]. Moreover, they tend to develop obesity‐related diseases at a younger age and at lower BMI thresholds than Europids [6, 7]. The mechanisms underlying their increased risk to develop T2DM are only partly known. South Asians typically exhibit an unfavourable metabolic phenotype characterized by higher fat mass, especially in visceral and ectopic areas, lower skeletal muscle mass, and dyslipidemia [8]. Notably, markers of insulin resistance are already detectable at birth in this population, suggesting early‐life programming of metabolic dysfunction [9, 10, 11, 12]. Furthermore, South Asians have lower resting energy expenditure, which may further contribute to increased fat storage [13].

Multiple gastrointestinal and pancreatic hormones regulate energy balance and insulin sensitivity, yet their postprandial dynamics across ethnic groups remain poorly understood. Incretin hormones such as glucagon‐like peptide‐1 (GLP‐1) and glucose‐dependent insulinotropic polypeptide (GIP) play key roles in postprandial glucose regulation by stimulating glucose‐dependent insulin secretion by pancreatic beta cells [14, 15, 16, 17]. Both GLP‐1 and GIP also act centrally to induce satiety [15, 17, 18]. Conversely, glucagon, secreted from pancreatic alpha cells, raises plasma glucose and increases energy expenditure [19]. Notably, recent therapeutic strategies for obesity and T2DM have leveraged the combined potential of GLP‐1, GIP, and glucagon to concomitantly induce satiety and increase energy expenditure [20]. Investigating the postprandial release of these hormones in South Asians versus Europids may uncover mechanisms that contribute to the higher cardiometabolic disease risk in South Asians and inform ethnic‐specific efficacy of incretin‐ or glucagon‐based therapies.

The mixed meal tolerance test (MMTT), a well‐established method that captures the integrated metabolic response to a nutrient‐rich stimulus, offers a physiologically relevant alternative to the oral glucose tolerance test (OGTT) [21, 22, 23]. The shape of the glucose response curve during an OGTT is associated with metabolic health [24]. Subjects with a monophasic OGTT glucose response curve (defined by a gradual increase in glucose levels between 30 and 90 min, followed by a peak and subsequent decrease in glucose of ≥ 4.5 mg/dL) have less beneficial markers of glucose homeostasis compared to those with a biphasic curve (defined by a second rise in glucose levels of ≥ 4.5 mg/dL after a decline of the initial rise in glucose levels) and an increased risk to develop T2DM in the future [25, 26, 27]. Studies on glucose curve morphology following a MMTT are more scarce, and the clinical implications of glucose curve morphology remain less well established.

In the current study, we aimed to investigate the effect of an extended MMTT (up to 240 min) on postprandial excursion of GLP‐1, GIP, and glucagon, alongside key metabolic parameters (i.e., glucose, insulin, lipids) in young and lean adults of South Asian and Europid descent. Based on the disadvantageous metabolic phenotype of South Asians, we hypothesized that postprandial excursions of GLP‐1, GIP and glucagon are lower in South Asians compared with Europids, both in males and females.

## 2 Methods

### 2.1 Study Design

This study uses biological samples and data obtained from the CAMI project (Elucidating the high cardiovascular disease risk in South Asians: focus on monocyte phenotype and incretin hormones), an observational study conducted at the Leiden University Medical Center between June and October 2023. The primary outcome of this secondary study was incremental area under the curve of GLP‐1, GIP, and glucagon. As this study was a secondary analysis, no formal power calculation was performed. The anticipated sample size was based on a previous study from our group in which insulin and GLP‐1 excursions during an OGTT differed between healthy lean South Asian and Europid men [28]. In that study, group sizes of *n* = 8 and *n* = 10 were sufficient to detect ethnic differences in these outcomes. Based on these findings, a sample size of 12 participants per group was considered sufficient for the present study.

The study was approved by the Medical Ethics Committee of the LUMC and conducted in accordance with the principles of the revised Declaration of Helsinki [29]. Written informed consent was obtained from all participants prior to inclusion. The clinical trial is registered at ClinicalTrial.gov (no. NCT05829018). The primary objective of the CAMI study was to compare immune cell composition between lean adult Dutch South Asians (hereinafter: ‘South Asians’) and BMI‐ and age‐matched Dutch Europids (hereinafter: ‘Europids’). The current manuscript addresses one of the study's secondary objectives.

### 2.2 Participants

A total of forty‐nine lean participants were included in the study, comprising South Asian males (*n* = 12) and females (*n* = 12), and Europid males (*n* = 13) and females (*n* = 12). Additional inclusion criteria were a body mass index (BMI) of 18–25 kg/m^2^ and an age of 18–30 years. One additional Europid male was included (resulting in 13 males instead of 12) due to a technical problem that occurred during sample collection for the study's primary endpoint in one Europid male.

South Asian ethnicity was defined as having all four grandparents from Surinam, Bangladesh, India, Nepal, Pakistan, Afghanistan, Bhutan, or Sri Lanka. Europid ethnicity was defined by having four grandparents of originally descent from Europe.

Exclusion criteria were the presence of any relevant chronic disease, use of medication known to influence glucose and/or lipid metabolism, abuse of alcohol or other substances, smoking, engagement in vigorous exercise (more than 3 times per week), and allergies to milk or soy.

### 2.3 Procedure

Participants were asked to withhold from vigorous physical activity for 48 h, and from consuming alcohol or caffeinated drinks for 24 h prior to the study visit. In addition, they were instructed to eat a standardized meal in the evening before the experiment and arrive fasted overnight.

### 2.4 Questionnaires and Anthropometric Measurements

Participants arrived at the facility at 08:00 am, where they underwent questionnaires about their medical history and current health. Thereafter, body weight and body composition were assessed using bioelectrical impedance analysis (BIA) (InBody720, InBody CO. Ltd., CA, USA). Height and waist and hip circumference were obtained using a measuring lint. BMI was calculated as weight in kilograms divided by height in meters squared (kg/m^2^).

### 2.5 Mixed Meal Tolerance Test

A catheter was inserted in the antecubital vein for venous blood sampling. An initial screening sample was obtained (Vacutainer SST II Advance Gel and EDTA tubes), to assess eligibility for inclusion. Screening parameters included full blood count, glucose, kidney function, liver enzymes, and lipid profile. Participants meeting the inclusion criteria proceeded with the study protocol. Prior to the MMTT, a baseline (−10 min) blood sample was obtained (Gel tubes, BD P800 collection tube, and EDTA tube). At approximately 9.00 am, participants ingested a standardized liquid meal (200 mL, 300 kcal, 36.8 g carbohydrates, 12.0 g protein, and 11.6 g fat; Nutridrink strawberry flavour, Nutricia), within 5 min. Subsequent blood samples were collected at 30, 60, 90, 120, 180, and 240 min post‐ingestion (7 time points total, including baseline). Blood samples collected in BD P800 and EDTA tubes were immediately placed on ice, while samples obtained in Gel tubes were allowed to clot for at least 30 min at room temperature. All samples were then centrifuged to obtain plasma or serum, respectively, and stored at −80°C until batch‐wise analyses. Plasma levels of total and active GLP‐1, total and active GIP, and glucagon were measured from blood collected in the BD P800 collection tubes using a U‐Plex Assay Platform (Meso‐Scale Diagnostics, Gaithersburg, MD, USA). Commercially available kits were used for the measurements of serum free fatty acids (FFA) (Wako chemicals, Neuss, Germany), serum triglycerides and serum total cholesterol (Roche Diagnostics, Woerden, the Netherlands), plasma glucose (Instruchemie, Delfzijl, the Netherlands), and serum insulin (Chrystal Chem, Elk Grove Village, IL, USA).

### 2.6 Statistical Analysis

Data are expressed as mean ± standard deviation. The normality of data was assessed using the Shapiro–Wilk test, visual histograms, and Q–Q plots. For the baseline characteristics, HOMA‐IR was calculated by multiplying fasting insulin levels (mU/L) with fasting glucose levels (mmol/L) and dividing this by 22.5. Glucose curves were defined as either monophasic of biphasic as follows as described previously [25]: a monophasic response was defined as a gradual increase in plasma glucose to a peak followed by a subsequent decline within the first 120 min. A biphasic response was defined as a gradual increase in plasma glucose to a peak, followed by a fall of ≥ 0.25 mmol/L, and then a second rise of ≥ 0.25 mmol/L within the first 120 min. Missing data were only caused by the inability to retrieve blood samples at some time points during the MMTT. In case of missing data, analyses were performed on the available data. No imputation was performed due to the relatively small sample size.

Baseline characteristics of the participants were compared between ethnicities within the same sex using an independent *t*‐test for normally distributed data. Not normally distributed data were log 10 transformed to yield normal distribution (i.e., total cholesterol) and analysed using an independent *t*‐test. Non‐parametric tests were performed on data that were not normally distributed even after log 10 transformation (i.e., waist circumference, waist‐hip ratio, body fat percentage, fasting insulin, HOMA‐IR, and triglycerides).

For the comparison of the excursion of GLP‐1, GIP, and parameters for glucose and lipid metabolism in response to a MMTT, we calculated the total area under the curve (tAUC_0–240_, and based on the shape of the curves performed post hoc measurements of tAUC_0–60_ and tAUC_60–240_) with the trapezoid rule [30]. To determine the incremental AUC (iAUC_0–240_, and also post hoc measurements of iAUC_0–60_, and iAUC_60–240_), we subtracted the area below the baseline value from the tAUC_0–240_ using the net iAUC approach. Specifically, tAUC was calculated for each time interval, after which the corresponding baseline area (baseline concentration × time interval) was subtracted. To compare the tAUC and iAUC between the two ethnicities, the Mann–Whitney U test was used, as not all the data were normally distributed. In addition, we used a two‐way repeated measures ANOVA with the within‐subject factor ‘time’ and the between‐subject factor ‘ethnicity’ for the comparison of the response of various hormones throughout a mixed meal test between ethnicities. Sphericity was assessed using Mauchly's test; when the assumption was not met (*p* < 0.05), Greenhouse–Geisser–corrected degrees of freedom and *p*‐values were reported, otherwise sphericity‐assumed results were used. We compared the means of each time point between ethnicities of the general linear model using the estimated marginal means comparison corrected with the Bonferroni methods to correct for multiple tests.

All statistical analyses were performed using SPSS v.29.0.1.0. Armonk, NY: IBM Corp. All graphs were created with GraphPad Prism software version 9.3.1 for Windows. Significance was set at *p* < 0.05.

## 3 Results

### 3.1 Baseline Characteristics

All groups were comparable with respect to age. South Asian males and females were shorter than their Europid counterparts (Table 1). Among males, there was no difference in body weight between ethnicities, which resulted in a higher BMI in South Asians. South Asian females had a lower body weight compared to Europid females, which combined with the shorter stature resulted in a similar BMI. The South Asian females had a lower lean mass compared to the Europid females. Furthermore, the South Asian males had a higher fat mass, and both South Asian males and females had a higher body fat percentage compared to the Europids. Fasting glucose, insulin and HOMA‐IR did not differ between ethnicities. Lastly, the South Asian males had a higher serum total cholesterol compared to Europid males.

**TABLE 1:** Baseline characteristics.

	Males		Females	
	Europids (*n* = 13)	South Asians (*n* = 12)	Europids (*n* = 12)	South Asians (*n* = 12)
Age, years	21.7 ± 2.9	23.3 ± 3.2	23.1 ± 2.1	23.3 ± 3.3
Body length, m	1.86 ± 0.07	1.79 ± 0.06*	1.74 ± 0.08	1.63 ± 0.06**
Body weight, kg	73.6 ± 6.0	74.9 ± 7.2	68.1 ± 9.1	60.3 ± 5.7*
BMI, kg/m^2^	21.3 ± 1.5	23.3 ± 1.5**	22.5 ± 1.2	22.6 ± 1.8
Waist circumference, cm	69.9 ± 12.9	75.7 ± 5.9	69.8 ± 4.6	68.9 ± 3.8
Hip circumference, cm	93.3 ± 3.1	94.1 ± 5.0	89.8 ± 6.4	87.9 ± 5.9
Waist to hip ratio	0.7 ± 0.1	0.8 ± 0.0	0.8 ± 0.0	0.8 ± 0.0
Lean mass, kg	66.4 ± 5.3	61.4 ± 8.2	51.5 ± 6.6	41.2 ± 3.6***
Fat mass, kg	7.3 ± 2.0	13.5 ± 5.5**	16.6 ± 6.1	19.1 ± 4.6
Fat percentage, %	9.8 ± 2.3	18.0 ± 7.2***	24.1 ± 6.5	31.5 ± 5.9*
Use of hormonal contraceptives, *n* (%)	n.a.	n.a.	6 (50%)	3 (25%)
Fasting glucose, mmol/L	4.9 ± 0.5	4.9 ± 0.3	4.8 ± 0.3	4.8 ± 0.3
Fasting insulin, mU/L	4.0 ± 2.0	4.4 ± 1.8	4.4 ± 1.6	4.4 ± 3.3
HOMA‐IR	0.8 ± 0.5	0.8 ± 0.5	0.9 ± 0.4	0.9 ± 0.7
Serum Total Cholesterol, mmol/L	3.1 ± 0.4	3.6 ± 0.5*	3.4 ± 0.5	3.7 ± 0.9
Serum Triglycerides, mmol/L	0.6 ± 0.2	0.8 ± 0.7	0.5 ± 0.2	0.6 ± 0.3

*Note:* Asterisk signs (*) indicate significant differences between ethnicities within a specific sex. *p < 0.05, **p < 0.01, ***p < 0.001.

Abbreviations: BMI, Body Mass Index; HOMA‐IR, Homeostatic Model Assessment for Insulin Resistance; n.a., not applicable.

### 3.2 South Asians Exhibit Biphasic Glucose Excursions During an MMTT

**FIGURE 1:**
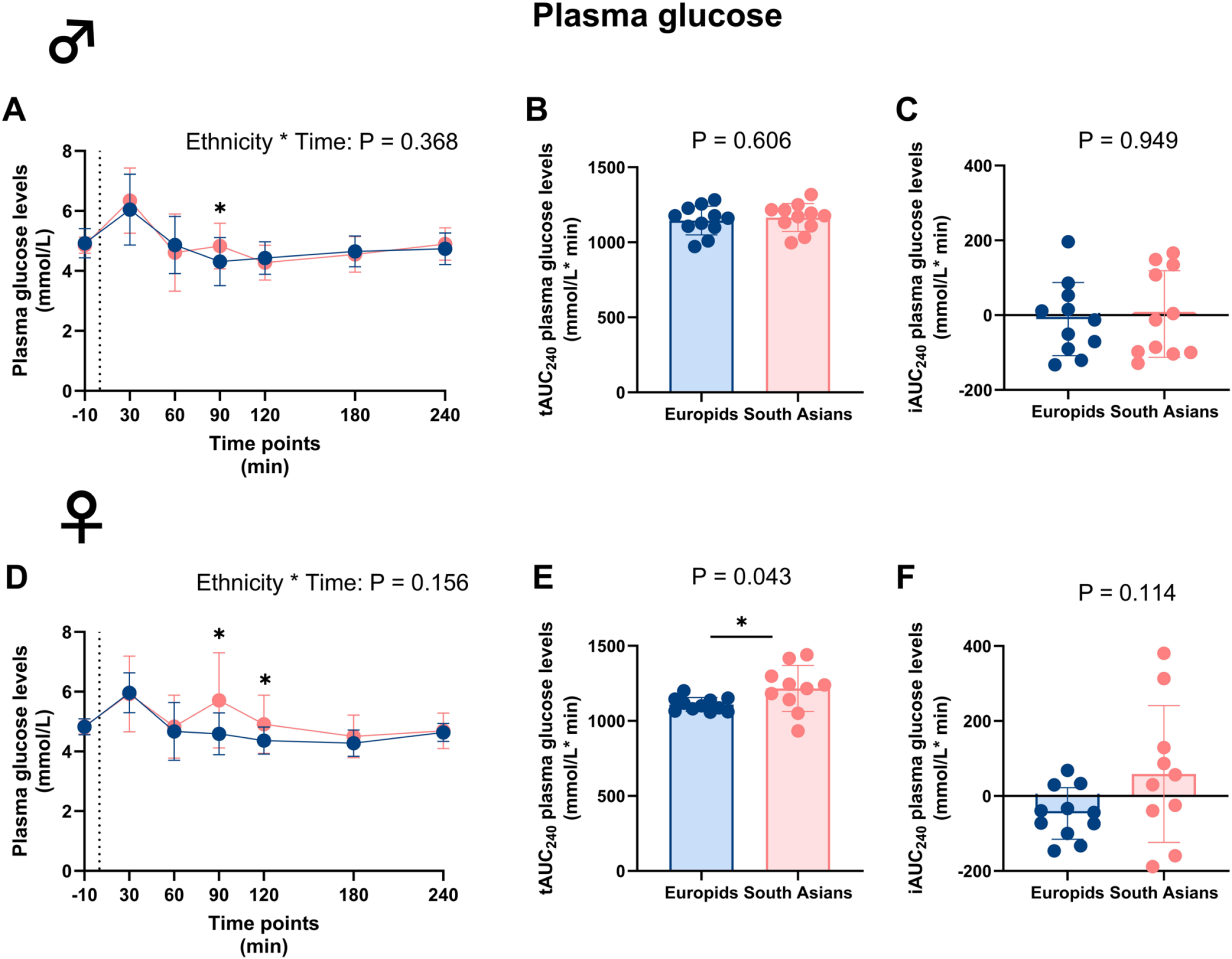
Plasma glucose levels before and during a mixed meal tolerance test in South Asian and Europid males and females. Line graphs showing plasma glucose levels before and during a mixed meal tolerance test (MMTT) in South Asian (*n* = 11) compared to Europid (*n* = 11) males (A). Box plots showing total area under the curve (tAUC_0–240_) (B) and incremental area under the curve (iAUC_0–240_) (C) in South Asian and Europid males. Similarly, line graphs showing plasma glucose levels during an MMTT in South Asian females (*n* = 10) compared to Europid females (*n* = 11) (D) and box plots showing tAUC_0–240_ now and iAUC_0–240_ (F) for South Asian and Europid females (F). Circles represent means in A and D and individuals' values in B, C, E, and F, and deviations are the standard deviations. Blue circles, lines, and boxes represent Europids, and pink circles, lines, and boxes represent South Asians. Dotted lines represent the time of the ingestion of the liquid meal (t = 0). We were unable to retrieve a blood sample of one South Asian male at two time points, and from one Europid male, two South Asian females, and one Europid female at one time point. *p < 0.05.

Following the mixed meal, South Asian and Europid males and females showed a peak in circulating plasma glucose levels after 30 min (Figure 1). Interestingly, South Asian males and females exhibited an additional glucose peak at 90 min, with glucose levels being significantly higher compared to both Europid males and females which pursued until 120 min in South Asian females. When assessing individual glucose curves, we found that 7 out of 12 Europid females (58%) had a biphasic curve and 6 out of 12 South Asian females (50%). Of note, 4 out of 13 Europid males (31%) had a biphasic curve compared with 7 out of 12 South Asian males (58%), which tended to be higher (*p* = 0.17) in the South Asian males. Although the changes in glucose curve did not result in significant differences over time between the ethnicities (Figure 1, Tables S1 and S2), this biphasic response led to a significantly elevated tAUC_0–240_ of the glucose excursion in South Asian compared to Europid females (Table S2 and Figure 1). After evaluating both peaks individually and calculating tAUC_0–60_ and tAUC_60–240_ of the glucose response, only tAUC_60–240_ was significantly higher in South Asian compared to Europid females (Figure S1). Furthermore, the iAUC_0–240_ for the glucose excursion did not differ between ethnicities in either sex (Tables S1 and S2 and Figure 1).

**FIGURE 2:**
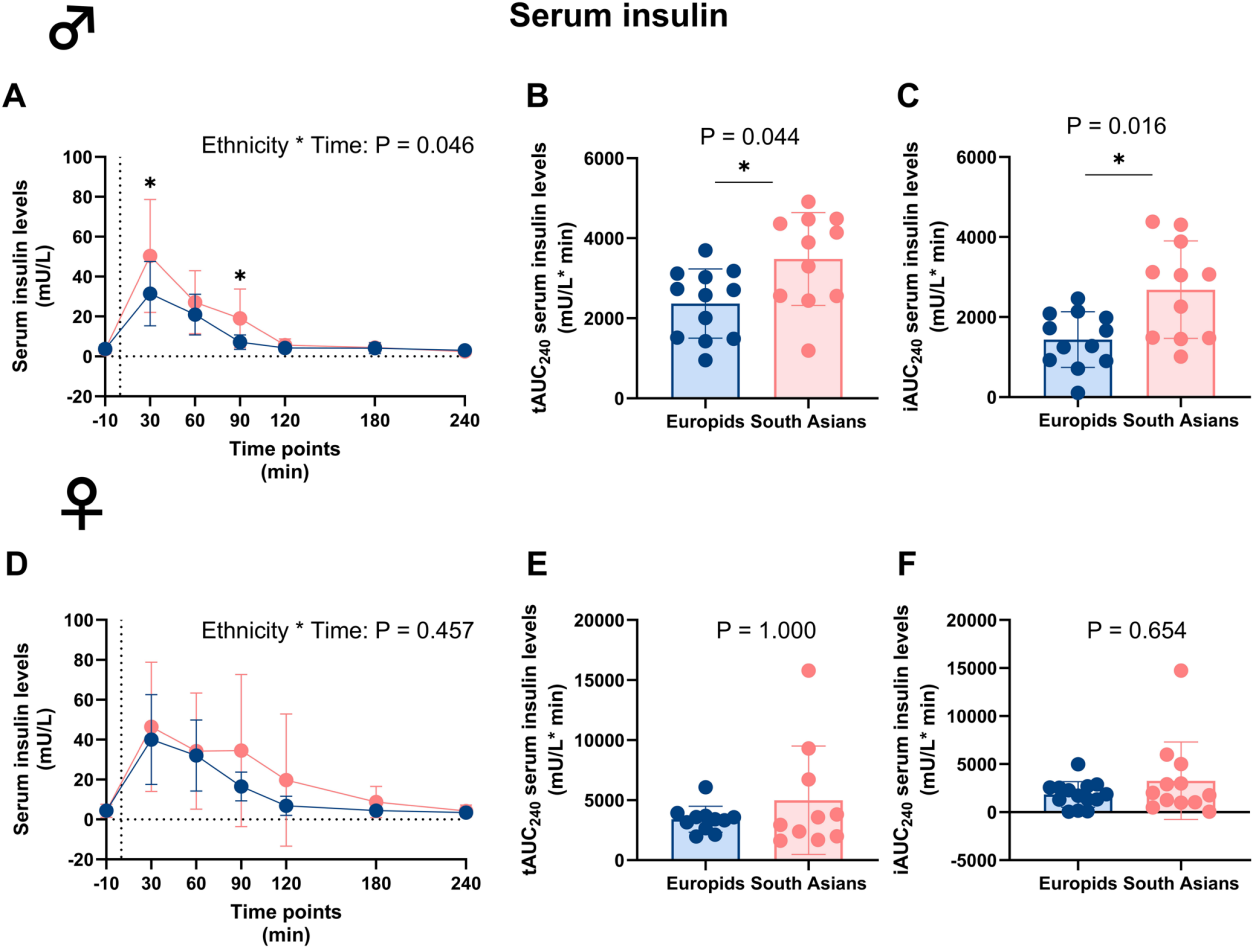
Serum insulin levels before and during a mixed meal tolerance test in South Asian and Europid males and females. Line graphs showing serum insulin levels before and during a mixed meal tolerance test (MMTT) in South Asian (*n* = 11) compared to Europid (*n* = 12) males (A). Box plots showing the total area under the curve (tAUC_0–240_) (B) and incremental area under the curve (iAUC_0–240_) (C) in South Asian and Europid males. Similarly, line graphs showing serum insulin levels during an MMTT in South Asian females (*n* = 10) compared to Europid females (*n* = 11) (D) and box plots showing the tAUC_0–240_ (E) and iAUC_0–240_ (F) for South Asian and Europid females (F). Circles represent means in A and D, and individuals' values in B, C, E, and F, and deviations are the standard deviations. Blue circles, lines, and boxes represent Europids, and pink circles, lines, and boxes represent South Asians. Dotted lines represent the time of the ingestion of the liquid meal (t = 0). We were unable to retrieve a blood sample of one South Asian female at two time points, and from one South Asian male, Europid male, one South Asian female, and one Europid female at one time point. *p < 0.05.

In South Asian males, serum insulin levels were higher compared to Europid males at 30 and 90 min (Figure 2). This resulted in significantly different levels of circulating serum insulin over time, a higher tAUC_0–240_, and a higher iAUC_0–240_ in South Asian compared to Europid males (Table S1 and Figure 2) but not females (Table S2 and Figure 2). For males and females, both the tAUC_0–60_ and the tAUC_60–240_ for the insulin excursion was not different between South Asians and Europids (Figure S2).

### 3.3 South Asian Females Have Lower Circulating Plasma Glucagon Levels at 90 min After MMTT Compared to Europid Females

**FIGURE 3:**
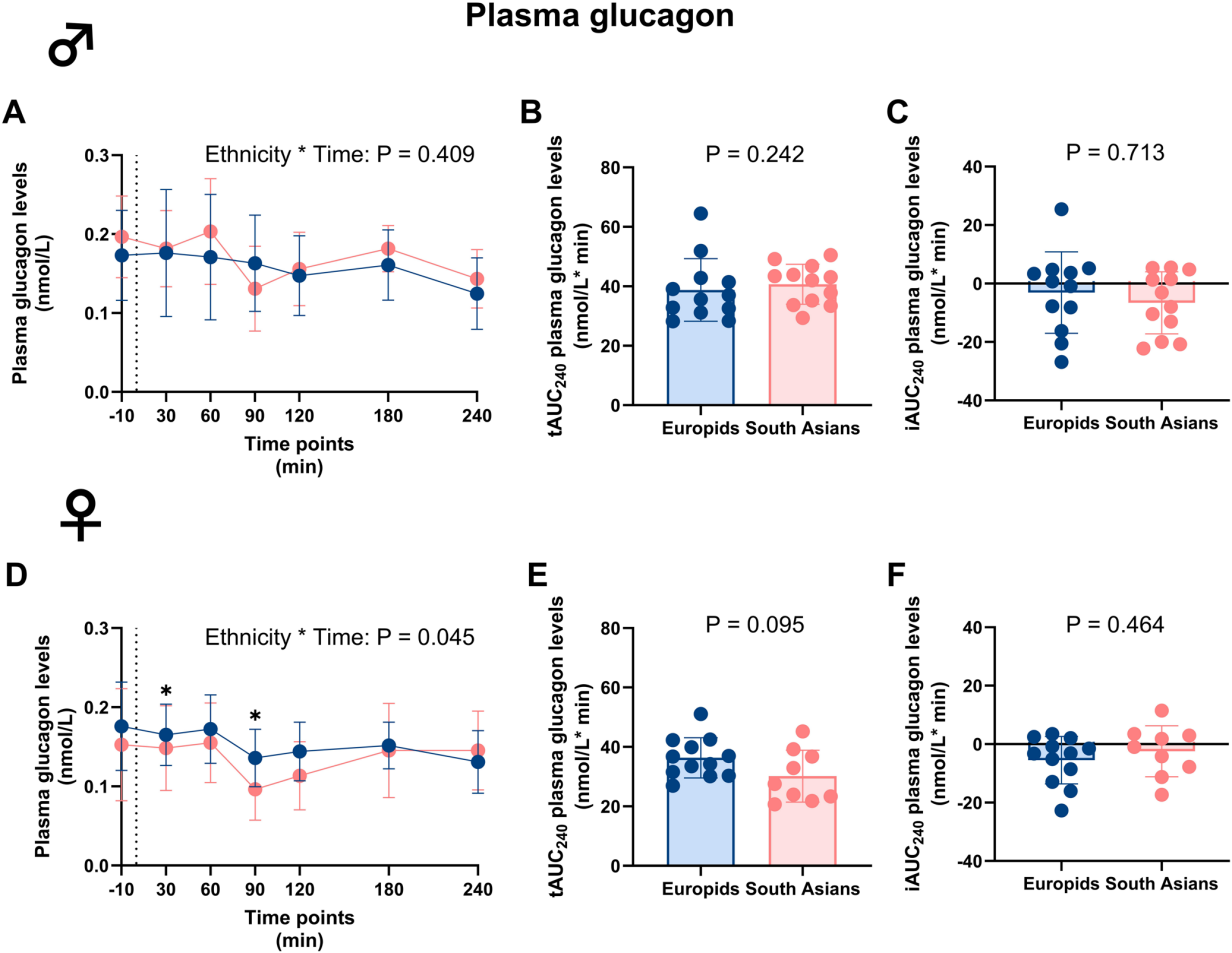
Plasma glucagon levels before and during a mixed meal tolerance test in South Asian and Europid males and females. Line graphs showing plasma glucagon levels before and during a mixed meal tolerance test (MMTT) in South Asian (*n* = 12) compared to Europid (*n* = 12) males (A). Box plots showing the total area under the curve (tAUC_0–240_) (B) and incremental area under the curve (iAUC_0–240_) (C) in South Asian and Europid males. Similarly, line graphs showing plasma glucagon levels during an MMTT in South Asian females (*n* = 9) compared to Europid females (*n* = 12) (D) and box plots showing the tAUC_0–240_ (E) and iAUC_0–240_ (F) for South Asian and Europid females (F). Circles represent means in A and D, and individuals' values in B, C, E, and F, and deviations are the standard deviations. Blue circles, lines, and boxes represent Europids, and pink circles, lines, and boxes represent South Asians. Dotted lines represent the time of the ingestion of the liquid meal (t = 0). Due to a technical error, one sample of one Europid male is missing, and we were unable to retrieve a blood sample and of three South Asian females at one time point. *p < 0.05.

In males, we did not find any difference in excursion of circulating plasma glucagon levels between South Asians and Europids over time. Correspondingly, tAUC_0–240_ and iAUC_0–240_ did not differ (Table S1 and Figure 3). In females, however, South Asians exhibited lower circulating plasma glucagon levels at 30 and 90 min (Figure 3). This resulted into significant differences in circulating plasma glucagon levels over time during the MMTT between South Asian and Europid females (P_Interaction_ = 0.045, Table S2 and Figure 3). The tAUC_0–240_ tended to be lower in South Asian compared to Europid females (Table S2 and Figure 3, while tAUC_0–60_ or tAUC_60–240_ for the glucagon excursion were not different; Figure S3). Furthermore, iAUC_0–240_ did not differ between ethnicities in females.

### 3.4 South Asian Males Exhibit Lower Circulating Plasma Total GLP‐1 Levels While Females Exhibit Higher Active GLP‐1 During an MMTT Compared to Europids

**FIGURE 4:**
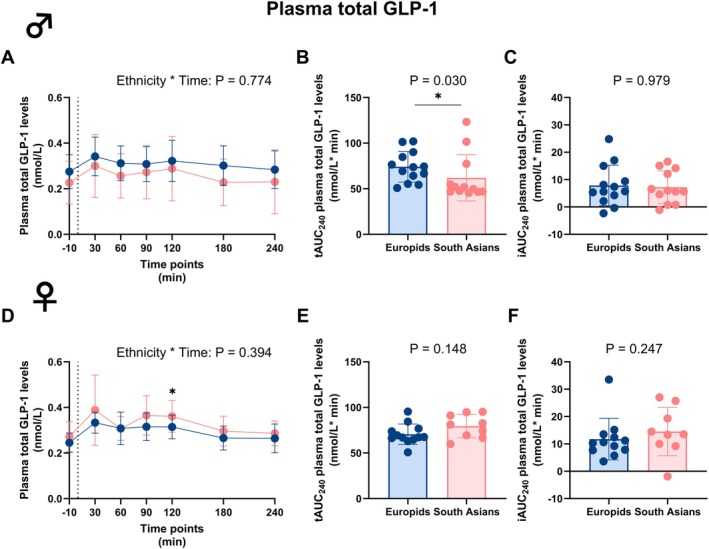
Plasma total glucagon‐like peptide‐1 levels before and during a mixed meal tolerance test in South Asian and Europid males and females. Line graphs showing plasma total glucagon‐like peptide‐1 (GLP‐1) levels before and during a mixed meal tolerance test (MMTT) in South Asian (*n* = 12) compared to Europid (*n* = 13) males (A). Box plots showing the total area under the curve (tAUC_0–240_) (B) and incremental area under the curve (iAUC_0–240_) (C) in South Asian and Europid males. Similarly, line graphs showing the plasma GLP‐1 levels during an MMTT in South Asian females (*n* = 9) compared to Europid females (*n* = 12) (D) and box plots showing the tAUC_0–240_ (E) and iAUC_0–240_ (F) for South Asian and Europid females (F). Circles represent means in A and D, and individuals' values in B, C, E, and F, and deviations are the standard deviations. Blue circles, lines, and boxes represent Europids, and pink circles, lines, and boxes represent South Asians. Dotted lines represent the time of the ingestion of the liquid meal (t = 0). We were unable to retrieve a blood sample from three South Asian females at one time point. *p < 0.05.

**FIGURE 5:**
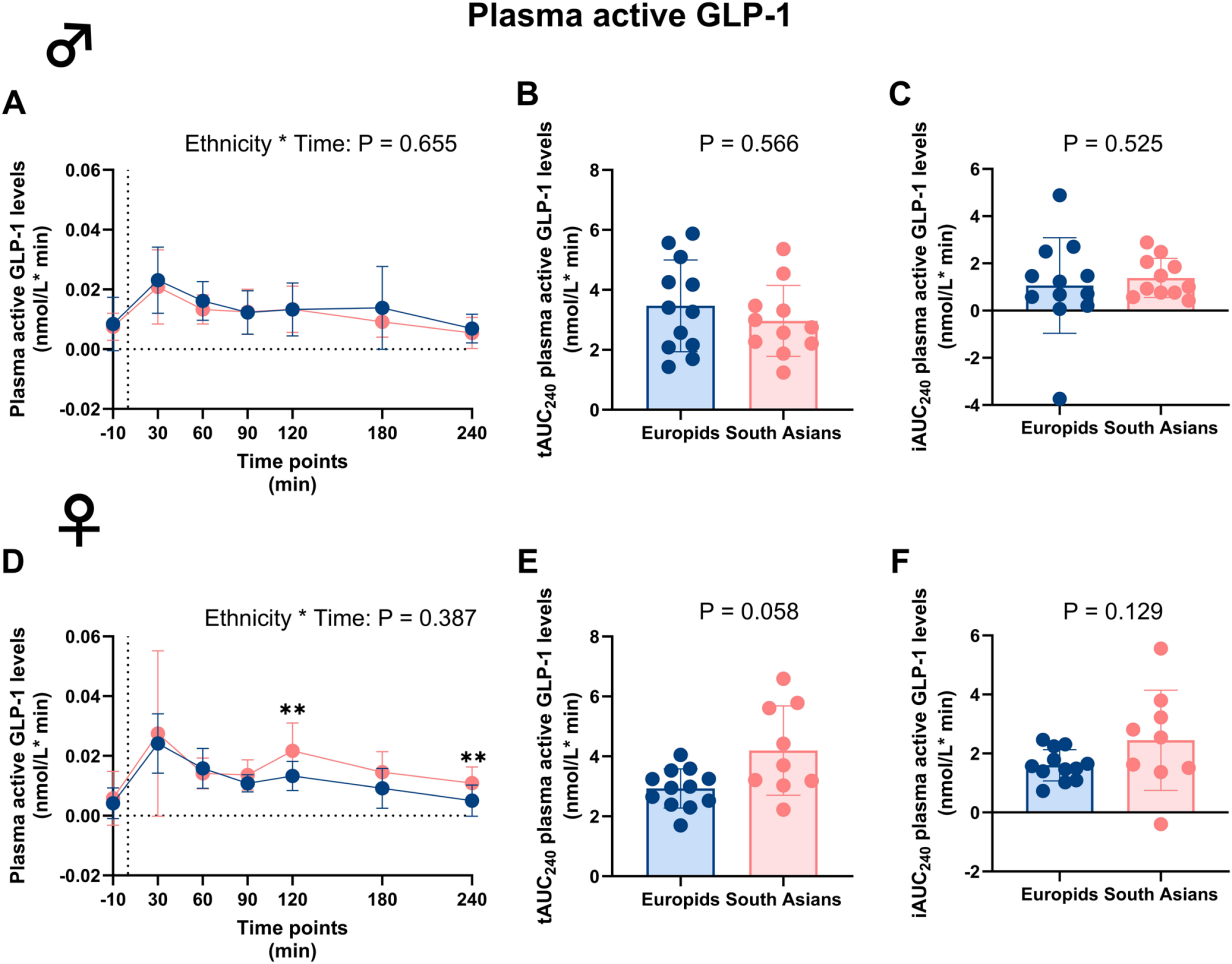
Plasma active glucagon‐like peptide‐1 levels before and during a mixed meal tolerance test in South Asian and Europid males and females. Line graphs showing plasma active glucagon‐like peptide‐1 (GLP‐1) levels before and during a mixed meal tolerance test (MMTT) in South Asian (*n* = 12) compared to Europid (*n* = 13) males (A). Box plots showing the total area under the curve (tAUC_0–240_) (B) and incremental area under the curve (iAUC_0–240_) (C) in South Asian and Europid males. Similarly, line graphs showing the plasma active GLP‐1 levels during an MMTT in South Asian females (*n* = 9) compared to Europid females (*n* = 12) (D) and box plots showing the tAUC_0–240_ (E) and iAUC_0–240_ (F) for South Asian and Europid females (F). Circles represent means in A and D, and individuals' values in B, C, E, and F, and deviations are the standard deviations. Blue circles, lines, and boxes represent Europids, and pink circles, lines, and boxes represent South Asians. Dotted lines represent the time of the ingestion of the liquid meal (t = 0). We were unable to retrieve a blood sample of three South Asian females at one time point. **p < 0.01.

Next, we assessed the excursions of plasma incretins during the MMTT. In males, circulating plasma levels of both total GLP‐1 and active GLP‐1 were not different at different time points and over time between ethnicities (Figures 4 and 5). However, in South Asian males, the tAUC_0–240_ of circulating total GLP‐1, but not active GLP‐1, was significantly lower compared to Europids (Figures 4 and 5 and Table S1). For excursions of total GLP‐1, tAUC_0–60_ tended to be lower and tAUC_60–240_ was significantly lower in South Asian males compared to Europids (Figure S4). The iAUC_0–240_ for circulating total and active GLP‐1 excursions were not different in males in either ethnicity (Figures 4 and 5 and Table S1).

In females, South Asians had higher levels of circulating plasma total and active GLP‐1 at 120 min during the MMTT (Figures 4 and 5). Circulating plasma active GLP‐1 was also higher at 240 min in South Asian females compared to Europid females (Figure 5). However, this did not result in a different excursion of circulating plasma total and active GLP‐1 over time between ethnicities (Figures 4 and 5 and Table S2) or tAUC_0–240_ (and iAUC_0–240_ (Table S2, Figures 4 and 5)). Interestingly, the tAUC_60–240_ for active GLP‐1 was significantly higher in South Asian compared to Europid females (Figure S4).

### 3.5 South Asian Females Exhibit Higher Circulating Plasma Total and Active GIP Levels During an MMTT Compared to Europid Females

**FIGURE 6:**
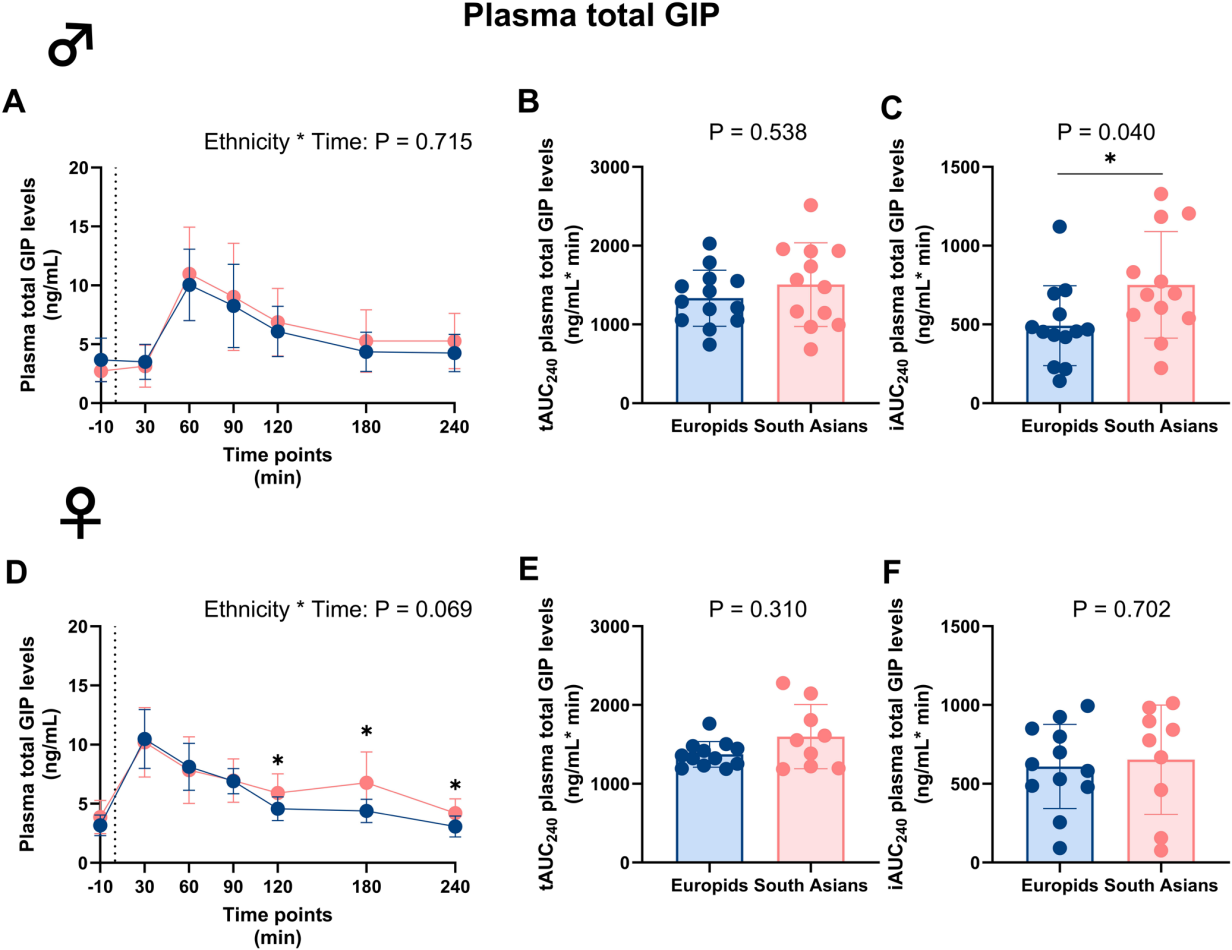
Plasma total glucose‐dependent insulinotropic polypeptide levels before and during a mixed meal tolerance test in South Asian and Europid males and females. Line graphs showing plasma total glucose‐dependent insulinotropic polypeptide (GIP) levels before and during a mixed meal tolerance test (MMTT) in South Asian (*n* = 12) compared to Europid (*n* = 13) males (A). Box plots showing the total area under the curve (tAUC_0–240_) (B) and incremental area under the curve (iAUC_0–240_) (C) in South Asian and Europid males. Similarly, line graphs showing the plasma GIP levels during an MMTT in South Asian females (*n* = 9) compared to Europid females (*n* = 12) (D) and box plots showing the tAUC_0–240_ (E) and iAUC_0–240_ (F) for South Asian and Europid females (F). Circles represent means in A and D, and individuals' values in B, C, E, and F, and deviations are the standard deviations. Blue circles, lines, and boxes represent Europids, and pink circles, lines, and boxes represent South Asians. Dotted lines represent the time of the ingestion of the liquid meal (t = 0). We were unable to retrieve a blood sample of three South Asian females at one time point. *p < 0.05.

In males, circulating plasma total and active GIP levels did not differ over time between both ethnicities (Table S1 and Figure 6). As a result, tAUC_0–240_ did not differ between ethnicities, also not for the tAUC_0–60_ and the tAUC_60–240_ (Figure S5). However, the iAUC_0–240_ was significantly higher in South Asian compared to Europid males.

**FIGURE 7:**
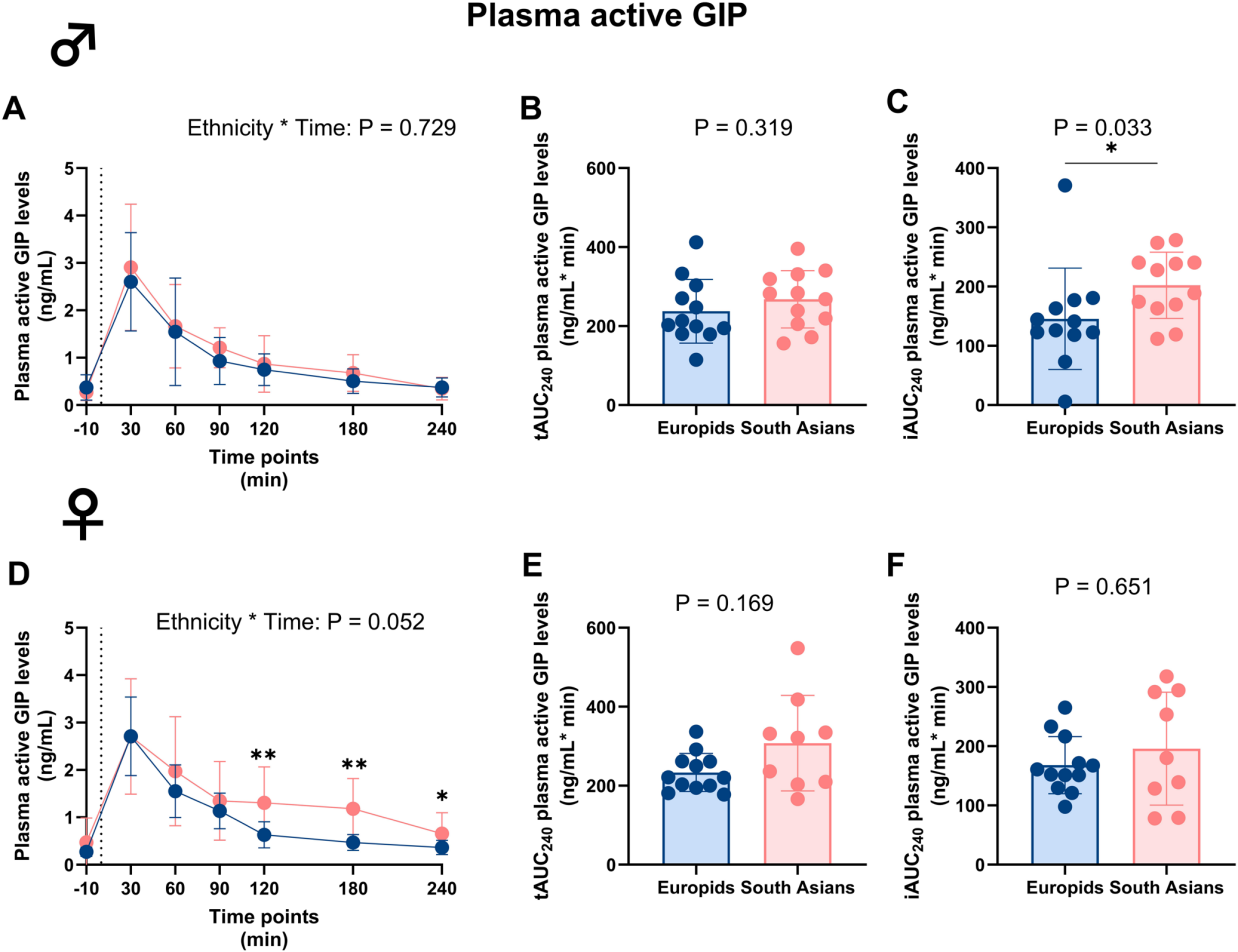
Plasma active glucose‐dependent insulinotropic polypeptide levels before and during a mixed meal tolerance test in South Asian and Europid males and females. Line graphs showing plasma active glucose‐dependent insulinotropic polypeptide (GIP) levels before and during a mixed meal tolerance test (MMTT) in South Asian (*n* = 12) compared to Europid (*n* = 12) males (A). Box plots showing the total area under the curve (tAUC_0–240_) (B) and incremental area under the curve (iAUC_0–240_) (C) in South Asian and Europid males. Similarly, line graphs showing the plasma active GIP levels during an MMTT in South Asian females (*n* = 9) compared to Europid females (*n* = 12) (D) and box plots showing the tAUC_0–240_ (E) and iAUC_0–240_ (F) for South Asian and Europid females (F). Circles represent means in A and D, and individuals' values in B, C, E, and F, and deviations are the standard deviations. Blue circles, lines, and boxes represent Europids, and pink circles, lines, and boxes represent South Asians. Dotted lines represent the time of the ingestion of the liquid meal (t = 0). We were unable to retrieve a blood sample of three South Asian females at one time point and of one Europid male; one time point is missing due to a technical failure. *p < 0.05, **p < 0.01.

In females, circulating plasma total and active GIP levels were higher in South Asians at 120, 180, and 240 min during the MMTT (Table S2 and Figures 6 and 7). This resulted in a tendency towards higher circulating plasma total GIP and active GIP levels over time (P_Interaction_ = 0.069 and P_Interaction_ = 0.052, respectively; Table S2, Figures 6 and 7), while the tAUC_0–240_ and iAUC_0–240_ of both circulating plasma total and active GIP did not differ in females between ethnicities. However, similarly to active GLP‐1 in South Asian females, plasma active GIP increased during the second part of the MMTT with a significantly higher tAUC_60–240_ of plasma active GIP (Figure S5).

### 3.6 Postprandial Lipid Excursions Do Not Differ Between South Asian and Europid Males and Females

Finally, we assessed serum lipid levels during the MMTT. In males, circulating FFA levels did not differ between time points or over time between South Asians and Europids (Table S1 and Figure S6). In females, circulating FFA levels remained suppressed longer over time in South Asians compared to Europids, which resulted in lower circulating FFA levels at 180 min and over time (P_Interaction_ = 0.022; Table S2 and Figure S6). While the tAUC_0–240_ of circulating FFA levels did not differ (Tables S1 and S2), the iAUC was lower in South Asians compared to Europids.

The responses of circulating triglyceride levels were similar in both South Asian males and females compared to their Europid counterparts (Tables S1 and S2 and Figure S6).

In males, circulating total cholesterol levels were higher in South Asians compared to Europids during all time points, resulting in no significant difference over time, but in a significantly higher tAUC_0–240_ in South Asian compared to Europid males, with no significant different iAUC_0–240_ (Table S1 and Figure S6). In females, circulating cholesterol levels remained similar between South Asians and Europids at the various time points and over time (Table S2 and Figure S6).

## 4 Discussion

In this study, we compared the excursions of GLP‐1, GIP, and glucagon, alongside markers of glucose and lipid metabolism, between young and lean South Asians and Europids in response to an extended MMTT. We observed notable ethnic differences in metabolic responses: South Asian males and females tended to exhibit a biphasic glucose excursion, contrasting the monophasic pattern seen in Europids. South Asian males exhibited an increased insulin response corresponding with the glucose peaks. South Asian females demonstrated higher active GLP‐1 and active GIP excursions during the second phase of the MMTT, and a tendency towards lower tAUC_0–60_ of circulating glucagon compared to Europid females. These findings suggest distinct incretin and glucoregulatory hormone dynamics between ethnicities that may result from biphasic gastric emptying in South Asians.

Firstly, in South Asians, we found a biphasic glucose response following the MMTT and more South Asians tended to follow this pattern compared to Europids. In healthy individuals, the circulating glucose levels in response to a meal or an oral glucose tolerance test often follow either a single peak or a biphasic curve, both of which are part of a normal physiological response [24, 31]. Supporting our findings, a recent study in healthy lean South Asian and Europid males [32] also reported a more pronounced biphasic glucose curve after a MMTT, suggesting ethnic differences in glucose excursion profiles. The precise clinical implication for this biphasic glucose pattern following a MMTT remains to be established, as most studies relating glucose morphology with metabolic phenotype and T2DM risk were done using an OGTT. This finding underscores that the physiological interpretation of glucose curve morphology may differ depending on the metabolic test used and the population studied.

We hypothesize that delayed gastric emptying rates in South Asians compared to Europids may partially underlie the observed biphasic glucose peak, consistent with reports of ethnic variation in gastric emptying rates between other ethnicities (e.g., Mexican Americans and American Indians compared to Europids) [33], although one study in older adults did not find an association between biphasic glucose excursion and gastric emptying rate [34]. Of note, participants in our study did not use any medication that affects gastric emptying rates, precluding this as a confounding factor. However, other factors –such as differences in β‐cell function, variable hepatic insulin extraction rates, incretin secretion, and incretin sensitivity– may also play a role in shaping the observed glucose excursion patterns. The clinical implications of the observed biphasic glucose curve for healthy South Asians and the possible development of T2DM later in life remain unknown and warrant further research. However, considering the well‐established link between elevated glucose levels and cardiometabolic diseases, the enhanced glucose tAUC_0–240_ that accompanied the biphasic glucose excursion in South Asians might already contribute to an enhanced T2DM risk.

The biphasic glucose response observed in South Asian females during the MMTT appears to coincide with distinct incretin dynamics. Specifically, the delayed second‐phase rise in active GLP‐1 and GIP from 90 min onward parallels the prolonged elevation in plasma glucose (tAUC_60–240_) observed in this group and may be a consequence of that. However, these results derived from an exploratory post hoc analysis should therefore be interpreted with caution. Despite these elevated incretin levels, insulin responses were not significantly different between South Asian and Europid females. The effectiveness of incretin hormones relies not only on their circulating concentrations but also on target tissue sensitivity, which cannot be assessed in this study. However, reduced insulin responses and slightly elevated glucose levels may suggest a diminished incretin effect in this group. As such, reduced incretin responsiveness may further contribute to the prolonged hyperglycemia observed in South Asian females, despite elevated hormone levels.

Our finding of higher postprandial GLP‐1 levels in South Asian females is in line with a previous study executed in lean Dutch South Asians compared to Dutch Europid males, in which a higher GLP‐1 peak was found in the South Asian men following an oral glucose tolerance test [27]. However, in that study, only a single increased peak of GLP‐1 was found in South Asians, which may have been due to the fact that an oral glucose load rather than a mixed meal was used. Remarkably, in our study, plasma total GLP‐1 excursion was lower in the South Asian compared to Europid males. This could have been explained by the fact that baseline levels of total GLP‐1 seemed to be lower, which persisted throughout the MMTT, and may indicate a reduced release of GLP‐1 by the enteroendocrine L‐cells in response to the mixed meal. An important question is how this sex difference in GLP‐1 excursions can be explained between ethnicities. Potentially, South Asian males are more sensitive to the effects of GLP‐1 compared to females—as also suggested by their higher insulin levels and relatively lower glucose concentrations compared to females—and therefore require lower postprandial GLP‐1 levels. On the other hand, studies in Europids have provided circumstantial evidence for higher GLP‐1 sensitivity in females compared to males, as supported by the fact that females show more pronounced relative weight loss with GLP‐1 analogues compared with males [35, 36]. Exploring incretin‐based sex differences in the South Asian population is an interesting topic for future studies. Importantly, GIP also plays an important role in the release of insulin postprandially. Of note, although in males, total GIP levels were equal between South Asians and Europids, the iAUC_240_ was higher for both total and active GIP, supporting a steeper increase in GIP release during the MMTT. Indeed, for total GIP, this was especially evident between 30 and 60 min during the MMTT.

DPP4 is the enzyme that quickly breaks down circulating GLP‐1, thereby forming another regulation of GLP‐1 excursions. To the best of our knowledge, DPP4 activity has not been systematically compared between South Asians and other ethnic groups. Elevated DPP4 activity can lead to reduced levels of active incretin hormones, potentially diminishing their glucose‐lowering effects. Given that ethnic variations in DPP4 activity and genetic polymorphisms have been linked to differences in DPP4 inhibitor efficacy, it is plausible that similar genetic factors in South Asians may exist.

Insulin decreases glucagon levels to help maintain glucose homeostasis. However, despite the higher serum insulin excursion in South Asian males, we did not observe a significant difference in plasma glucagon levels compared to Europid males. In females, however, plasma glucagon was significantly lower in South Asians compared to Europids at 30 and 90 min. This is likely not driven by insulin levels, since we did not observe significantly higher serum insulin levels in this group. Potentially, the increased glucose levels that occurred as part of the biphasic glucose response in South Asian females could be contributing to lower glucagon levels by acting directly on the pancreatic alpha cells [37]. Given that glucagon increases energy expenditure [34], the lower plasma glucagon levels in South Asian females could contribute to the lower energy expenditure known in the South Asian population [13]. However, to our knowledge, differences in (postprandial) glucagon levels between South Asians and Europids and their relationship to variations in resting energy expenditure have not been previously reported.

Various incretin‐based pharmacological interventions are emerging for the treatment of obesity and T2DM, which improve insulin sensitivity and reduce nutrient intake by inducing satiety [38]. If the lower tAUC_0–240_ of total GLP‐1 levels observed in South Asian males in our study indeed impairs satiety, this may partly explain their increased vulnerability to weight gain and T2DM. Consequently, interventions targeting the GLP‐1‐axis could be especially beneficial for the South Asian population, especially in males. A previous study from our group showed similar metabolic benefits of the GLP‐1 receptor agonist liraglutide in South Asians and Europids with T2DM, including improved glycemic control and body composition [39, 40]. Interestingly, a recent meta‐analysis showed that GLP‐1 receptor agonists confer greater benefit in cardiovascular outcomes in Asians compared to whites [41].

A key strength of this study is the comprehensive measurement of multiple glucoregulatory, hunger and satiety hormones during the MMTT up to 240 min after food ingestion in both males and females. However, this study is not without limitations. Despite having a young, lean population with healthy BMIs, we already noticed metabolic differences. While matching these groups remains inherently challenging due to variations in fat mass, fat percentage, and potentially insulin sensitivity, these differences also reflect true physiological diversity rather than solely confounding factors. Furthermore, since this study was a secondary analysis, no heated blood samples were taken to mimic glucose metabolism from the arterial circulation [42]. Postprandial measurements of glucose and incretin hormones in venous blood samples may have been influenced by peripheral tissue extractions. We tried to overcome this as much as possible by letting participants rest on a bed during the MMTT to harmonize conditions. Furthermore, although the primary outcome of this secondary study was total iAUC of GLP‐1, GIP, and glucagon, multiple other analyses were performed. These should be interpreted as exploratory and serve as a basis for future confirmatory research.

## 5 Conclusion

South Asians exhibit a distinct metabolic response to an MMTT compared to Europids, characterized by a biphasic peak in glucose levels and, intriguingly, higher active GLP‐1 and active GIP levels late in the test—particularly in South Asian females. Our findings highlight that key metabolic hormones are uniquely regulated in South Asians compared to Europids, offering insights into the complex mechanisms underlying their heightened cardiometabolic risk.

## Funding

This work was supported by the Dutch Research Council NWO (VENI grant 09150161910073 to M.R.B.), by the Spanish Ministry of Science (RYC2022‐036473‐I; to B.M.T). We also thank Roba Metals B.V., IJsselstein, the Netherlands, for financial support.

## Disclosure

The authors have nothing to report.

## Conflicts of Interest

The authors declare no conflicts of interest.

## Supporting information


**Data S1:** Supporting Information.

## Data Availability

The datasets generated during and/or analysed during the current study are not publicly available but are available from the corresponding author on reasonable request.
