# Supplementary material for: GLP‐1, GIP, and Glucagon Excursions During a Mixed Meal Tolerance Test in Young and Lean South Asians Versus Europids
Source: Diabetes Obes Metab. 2026 Apr 15;28(6):5078–90. doi: 10.1111/dom.70704 (PMC13146191; doi:10.1111/dom.70704)
Supplement: Supplementary file 1 — Data S1: Supporting Information. [file DOM-28-5078-s001.docx]

Supplemental data to:

**GLP-1, GIP, and glucagon excursions during a mixed meal tolerance test in young and lean South Asian versus Europid males and females**

*Carlijn A. Hoekx^1,2^, Robin van Eenige^1,2^, Lisa B.D. Brinkman^1,2^, Sander Kooijman^1,2^, Marcel Muskiet^1,2^, Ingrid M. Jazet^1,2^, Borja Martinez-Tellez^1,2,3,4,5^, Patrick C.N. Rensen^1,2^, Mariëtte R. Boon^1,2,6,7^**

*1. Division of Endocrinology, Department of Medicine, Leiden University Medical Center, Leiden, The Netherlands*

*2. Einthoven Laboratory for Experimental Vascular Medicine, Leiden University Medical Center, Leiden, The Netherlands*

*3. Department of Nursing Physiotherapy and Medicine, SPORT Research Group (CTS-1024), CIBIS Research Center, University of Almería, Almería, Spain*

*4. Biomedical Research Unit, Torrecárdenas University Hospital, 04009 Almería, Spain*

*5. CIBER de Fisiopatología de la Obesidad y Nutrición (CIBEROBN), Instituto de Salud Carlos III, Granada, Spain*

*6. Obesity Center CGG, Erasmus MC, Rotterdam, the Netherlands*

*7. Division of Endocrinology, Department of Internal Medicine, Erasmus MC, Rotterdam, the Netherlands*

**Conflict of interest:** All authors declare that they have no conflict of interest.

***Corresponding author**: Mariëtte R. Boon, m.r.boon@lumc.nl, Leiden University Medical Center, Albinusdreef 2, 2333 ZA, Leiden, The Netherlands. ORCID ID: <https://orcid.org/0000-0002-3247-7538>

**Keywords:** Energy metabolism; South Asian; Mixed meal test, Incretin hormones

**Running title:** Effect of an MMTT on GLP-1, GIP, and glucagon in South Asians vs Europids

**Clinical trial registration:** ClinicalTrials.gov (NCT05829018; registration date: 25-04-2023)

**Supplemental Table 1. Overview of the area under the curve of the excursions of glucose, insulin, hormones, and lipids during a mixed meal tolerance test in South Asian compared to Europid males**

|  | **Europids** | | **South Asians** | |  | | |
| --- | --- | --- | --- | --- | --- | --- | --- |
|  | **Males** | | **Males** | | **P values** | | |
| Glycemic parameters | | | | | | | |
|  | **tAUC_0-240_** | **iAUC_0-240_** | **tAUC_0-240_** | **iAUC_0-240_** | **tAUC_0-240_** | **iAUC_0-240_** | **P_interaction_** |
| Plasma Glucose  (mmol/L * min) | 1145±99^c^ | 1107±94^c^ | 1165±93^c^ | 1126±94^c^ | 0.606^w^ | 0.949^w^ | 0.368^w^ |
| Serum Insulin  (mU/L * min) | 2369±865^b^ | 2338±855^b^ | 3483±1161^c^ | 3457±115^c^ | 0.044^x^ | 0.016^x^ | 0.046^x^ |
| Plasma Glucagon  (nmol/L * min) | 38.8±10.5^b^ | 37.4±10.4^b^ | 40.7±6.7^b^ | 39.1±6.5^b^ | 0.242^y^ | 0.713^y^ | 0.409^y^ |
| Incretin hormones | | | | | | | |
| Plasma Total GLP-1 (nmol/L * min) | 74.1±16.7^a^ | 71.9±16.2^a^ | 62.0±25.5^b^ | 60.2±24.7^b^ | 0.030^z^ | 0.979^z^ | 0.774^z^ |
| Plasma Active GLP-1 (nmol/L * min) | 3.5±1.5^b^ | 3.4±1.5^b^ | 3.0±1.2^c^ | 2.9±1.2^c^ | 0.566^x^ | 0.525^x^ | 0.655^x^ |
| Plasma Total GIP (ng/mL * min) | 1333±357^a^ | 1308±349^a^ | 1505±532^b^ | 1483±522^b^ | 0.538^z^ | 0.040^z^ | 0.715^z^ |
| Plasma Active GIP (ng/mL * min) | 238±81^c^ | 234±80^c^ | 268±72^c^ | 266±71^c^ | 0.319^w^ | 0.033^w^ | 0.660^w^ |
| Lipids | | | | | | | |
| Serum FFA (mmol/L * min) | 63.2±19.0^b^ | 59.3±17.6^b^ | 73.7±25.5^c^ | 69.7±24.0^c^ | 0.316^x^ | 0.651^x^ | 0.921^x^ |
| Serum TG (mmol/L * min) | 132.3±53.4^b^ | 128.3±52.1^b^ | 114.8±29.4^c^ | 110.9±28.6^c^ | 0.413^x^ | 0.169^x^ | 0.416^x^ |
| Serum TC (mmol/L * min) | 708±104^b^ | 683±101^b^ | 847±114^c^ | 819±110^c^ | 0.007^x^ | 0.059^x^ | 0.306^x^ |

*Table showing the mean and standard deviation of the total area under the curve (tAUC_0-240_) and incremental area under the curve (iAUC_0-240_) of the excursions of glucose, insulin, hormones, and lipids during a mixed meal tolerance test for both South Asian and Europid males. P-values of the comparisons between the two ethnicities were obtained via the non-parametric Man-Whitney U test and the p-values of the interactions were analyzed via a repeated measurement ANOVA. FFA, free fatty acids; GIP, glucose-dependent insulinotropic polypeptide; GLP-1, glucagon-like peptide-1; TC, total cholesterol; TG, triglycerides. Letters indicate n values of each ethnicity, ^a^n=13; ^b^n=12; ^c^n=11; ^d^n=10, ^e^n=9, and ^v^n=21; ^w^n=22; ^x^n=23; ^y^n=24, ^z^n=25.*

**Supplemental Table 2. Overview of the area under the curve of the excursion of glucose, insulin, hormones, and lipids during a mixed meal tolerance test in South Asian compared to Europid females**

|  | **Europids** | | **South Asians** | |  | | |
| --- | --- | --- | --- | --- | --- | --- | --- |
|  | **Females** | | **Females** | | **P values** | | |
| Glycemic parameters | | | | | | | |
|  | **TAUC_0-240_** | **iAUC_0-240_** | **TAUC_0-240_** | **IAUC_0-240_** | **TAUC_0-240_** | **iAUC_0-240_** | **P_int_** |
| Plasma Glucose  (mmol/L * min) | 1110±46^c^ | 1071±45^c^ | 1216±154^d^ | 1178±154^d^ | 0.043^v^ | 0.114^v^ | 0.156^v^ |
| Serum Insulin  (mU/L * min) | 3409±1089^c^ | 3373±1086^c^ | 4988±4519^d^ | 4951±4506^d^ | 1.000^v^ | 0.654^v^ | 0.457^v^ |
| Plasma Glucagon  (nmol/L * min) | 36.3±6.8^b^ | 35.0±6.4^b^ | 30.2±8.7^e^ | 29.1±8.3^e^ | 0.095^v^ | 0.464^v^ | 0.045^v^ |
| Incretin hormones and glucagon | | | | | | | |
| Plasma Total GLP-1 (nmol/L * min) | 70.5±11.3^b^ | 68.6±11.0^b^ | 79.5±12.8^e^ | 77.3±12.4^e^ | 0.148^v^ | 0.247^v^ | 0.394^v^ |
| Plasma Active GLP-1 (nmol/L * min) | 2.9±0.7^b^ | 2.9±0.6^b^ | 4.2±1.5^e^ | 4.1±1.5^e^ | 0.058^v^ | 0.129^v^ | 0.387^v^ |
| Plasma Total GIP (ng/mL * min) | 1374±164^b^ | 1351±164^b^ | 1598±408^e^ | 1571±402^e^ | 0.310^v^ | 0.702^v^ | 0.069^v^ |
| Plasma Active GIP (ng/mL * min) | 233±48^b^ | 231±48^b^ | 308±121^e^ | 304±118^e^ | 0.169^v^ | 0.651^v^ | 0.052^v^ |
| Lipids | | | | | | | |
| Serum FFA (mmol/L * min) | 94.6±25.1^c^ | 90.5±24.5^c^ | 84.2±30.0^d^ | 78.6±28.6^d^ | 0.349^v^ | 0.029^v^ | 0.022^v^ |
| Serum TG (mmol/L * min) | 137±45^c^ | 133±44^c^ | 170±72^d^ | 165±70^d^ | 0.314^v^ | 0.132^v^ | 0.538^v^ |
| Serum TC (mmol/L * min) | 805±129^c^ | 778±125^c^ | 850±195^d^ | 821±188^d^ | 0.654^v^ | 0.197^v^ | 0.454^v^ |

*Table showing the mean and standard deviation of the total area under the curve (tAUC_0-240_) and incremental area under the curve (iAUC_0-240_) of the excursions of glucose, insulin, hormones, and lipid during a mixed meal tolerance test for both South Asian and Europid females. P-values of the comparisons between the two ethnicities were obtained via the non-parametric Man-Whitney U test and the p-values of the interaction were analyzed via a repeated measurement ANOVA. FFA, free fatty acids; GIP, glucose-dependent insulinotropic polypeptide; GLP-1, glucagon-like peptide-1; TC, total cholesterol; TG, triglycerides. Letters indicate n values of each ethnicity, ^b^ n=12; ^c^n=11; ^d^n=10, ^e^n=9, and ^v^=21*


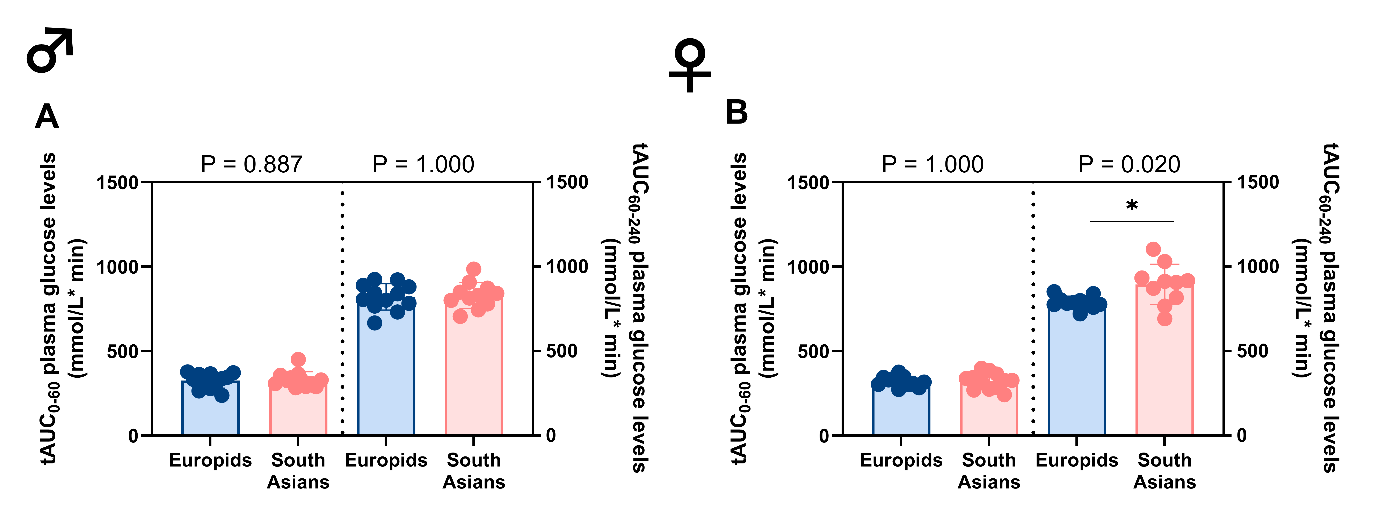


**Supplemental Figure 1. Total areas under the curve of the glucose excursion within two periods during the mixed meal tolerance test in South Asian and Europid males and females**

Box plots showing the total areas under the curve within two periods (tAUC_0-60_ and tAUC_60-240_) of the glucose excursion during the mixed meal tolerance test in South Asian (n=11) compared to Europid (n=12) males (**A)** and in South Asian (n=10) compared to Europid (n=11) females (**B**). Circles represent individuals’ values and deviations are the standard deviations. Blue circles, lines, and boxes represent Europids, and pink circles, lines, and boxes represent South Asians. We were unable to retrieve a blood sample of one South Asian male at two time points, and of one Europid male, two South Asian females, and one Europid female at one time point.


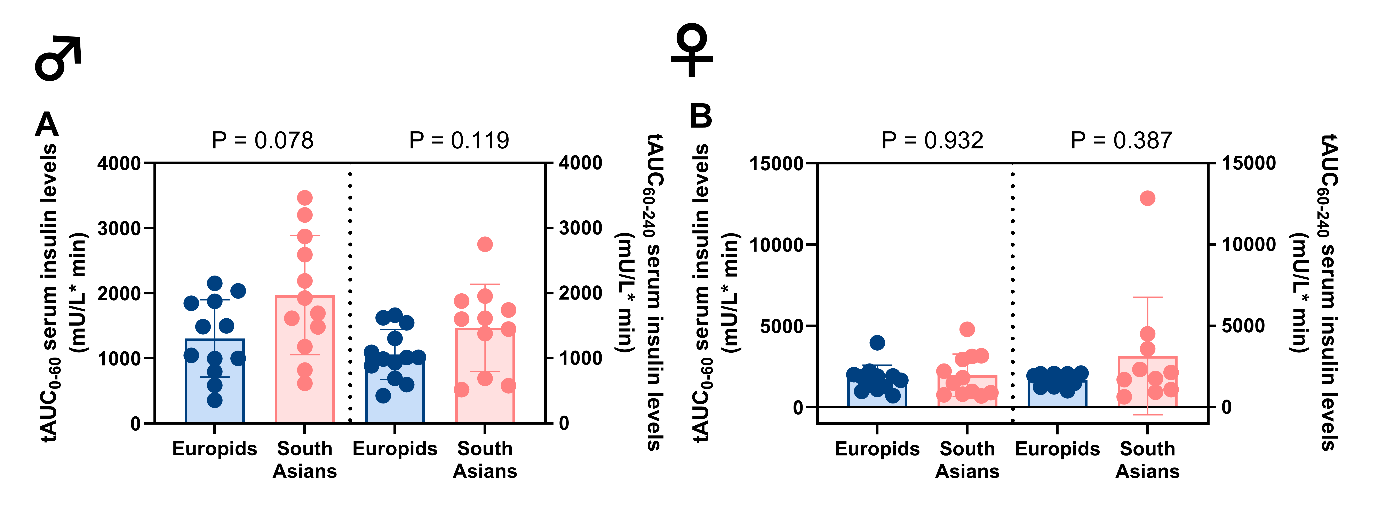


**Supplemental Figure 2. Total areas under the curve of the insulin excursion within two periods during the mixed meal tolerance test in South Asian and Europid males and females**

Box plots showing the total areas under the curve within two periods (tAUC_0-60_ and tAUC_60-240_) of the insulin excursion in South Asian (n=11) compared to Europid (n=12) males (**A)** and in South Asian (n=10) compared to Europid (n=11) females (**B**). Circles represent individuals’ values and deviations are the standard deviations. Blue circles, lines, and boxes represent Europids, and pink circles, lines, and boxes represent South Asians. We were unable to retrieve a blood sample of one South Asian female at two time points, and from one South Asian male, Europid male, one South Asian female, and one Europid female at one time point.


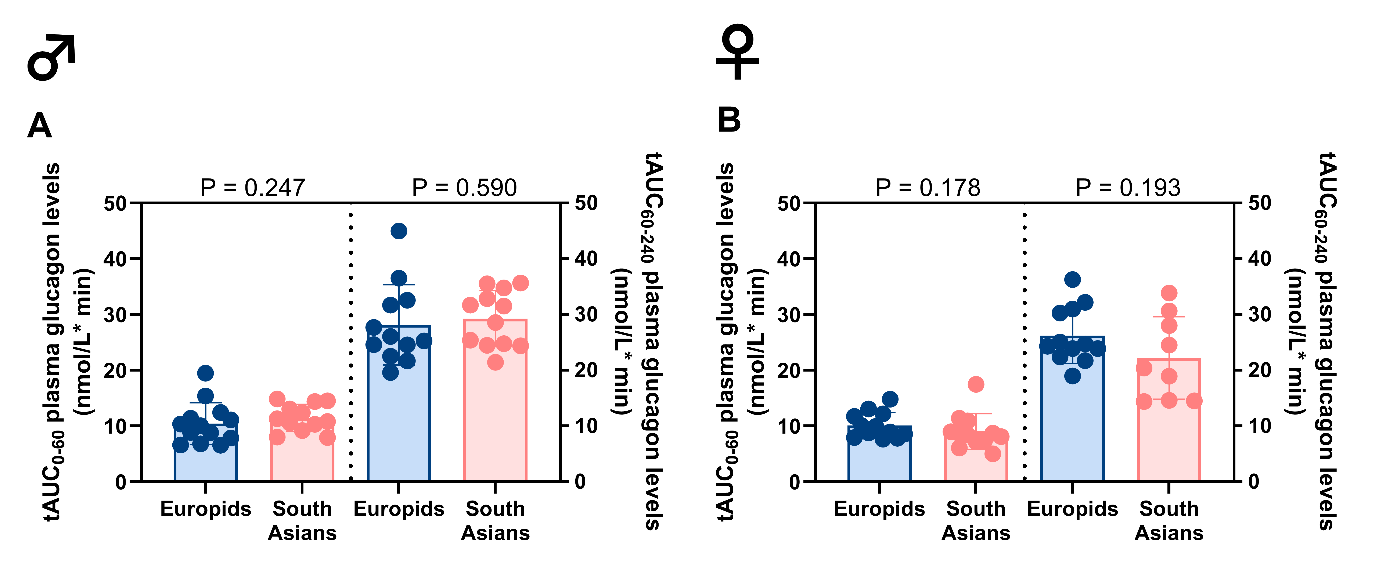


**Supplemental Figure 3. Total areas under the curve of the glucagon excursion within two periods during the mixed meal tolerance test in South Asian and Europid males and females**

Box plots showing the total areas under the curve within two periods (tAUC_0-60_ and tAUC_60-240_) of the glucagon excursion during the mixed meal tolerance test in South Asian (n=12) compared to Europid (n=12) males (**A)** and in South Asian (n=9) compared to Europid (n=12) females (**B**). Circles represent individuals’ values and deviations are the standard deviations. Blue circles, lines, and boxes represent Europids, and pink circles, lines, and boxes represent South Asians. Due to a technical error, one sample of one Europid male is missing and we were unable to retrieve a blood sample of three South Asian females at one time point.


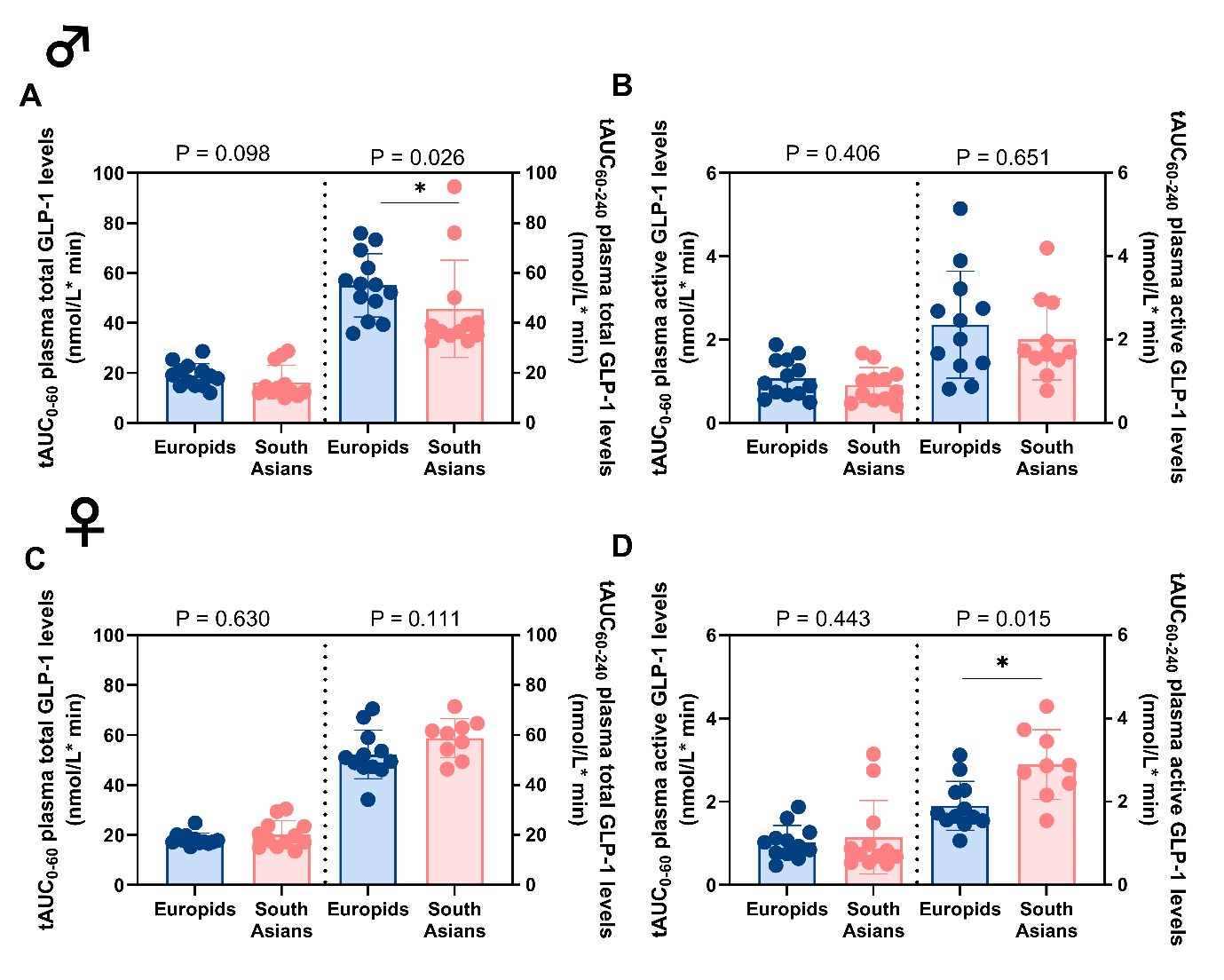


**Supplemental Figure 4. Total areas under the curve of the total and active glucagon-like peptide-1 excursions within two periods during the mixed meal tolerance test in South Asian and Europid males and females**

Box plots showing the total areas under the curve within two periods (tAUC_0-60_ and tAUC_60-240_) of the total glucagon-like peptide-1 (GLP-1) excursion in South Asian (n=12) compared to Europid (n=13) males (**A**) and box plots showing the tAUC_0-60_  and tAUC_60-240_ of active GLP-1 excursion in South Asian (n=11) and Europid (n=12) males (**B**). Box plots showing the tAUC_0-60_ and tAUC_60-240_ of the GLP-1 excursions in South Asian (n=9) compared to Europid (n=12) females (**C**) and box plots showing the tAUC_0-60_ and tAUC_60-240_ of active GLP-1 excursion in South Asian (n=9) and Europid (n=12) females (**D**). Circles represent individuals’ values and deviations are the standard deviations. Blue circles, lines, and boxes represent Europids, and pink circles, lines, and boxes represent South Asians. We were unable to retrieve a blood sample of one South Asian female at two time points, and from one South Asian male, Europid male, one South Asian female, and one Europid female at one time point.


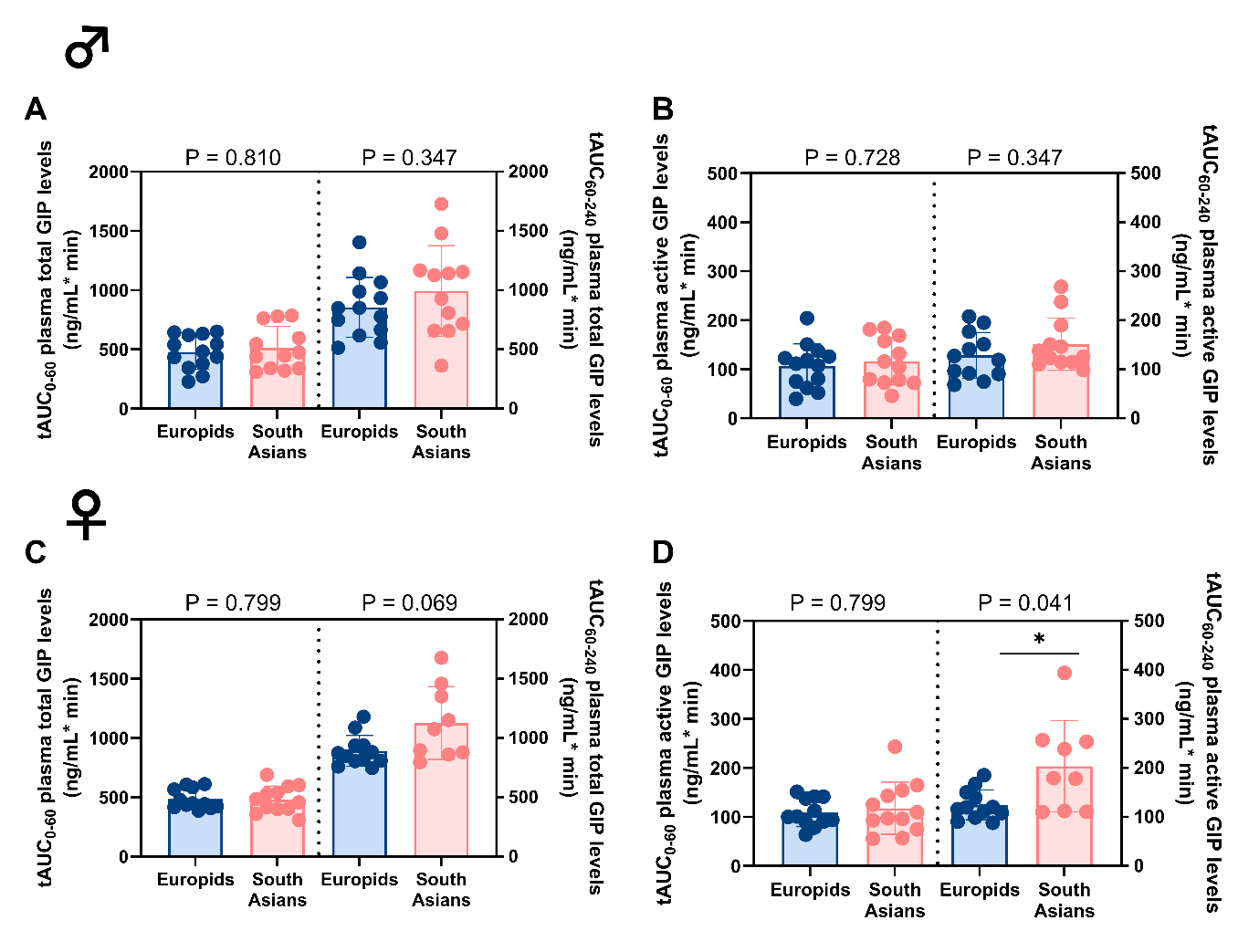


**Supplemental Figure 5. Total areas under the curve of total and active glucose-dependent insulinotropic polypeptide excursions within two periods during the mixed meal tolerance test in South Asian and Europid males and females**

Box plots showing the total areas under the curve within two periods (tAUC_0-60_ and tAUC_60-240_) of the total glucose-dependent insulinotropic polypeptide (GIP) excursions during the mixed meal tolerance test (MMTT) in South Asian (n=12) compared to Europid (n=13) males (**A**) and box plots showing the tAUC_0-60_ and tAUC_60-240_ of active GIP excursions in South Asian (n=12) and Europid males (n=12) (**B**). Box plots showing tAUC_0-60_ and tAUC_60-240_ of total GIP excursion during the MMTT in South Asian (n=9) compared to Europid (n=12) females (**C**) and box plots showing tAUC_0-60_ and tAUC_60-240_ of active GIP excursions in South Asian (n=9) and Europid (n=12) females (**D**). Circles represent individuals’ values and deviations are the standard deviations. Blue circles, lines, and boxes represent Europids, and pink circles, lines, and boxes represent South Asians. We were unable to retrieve a blood sample of three South Asian females at one time point and of one Europid male one time point missing due to a technical failure.


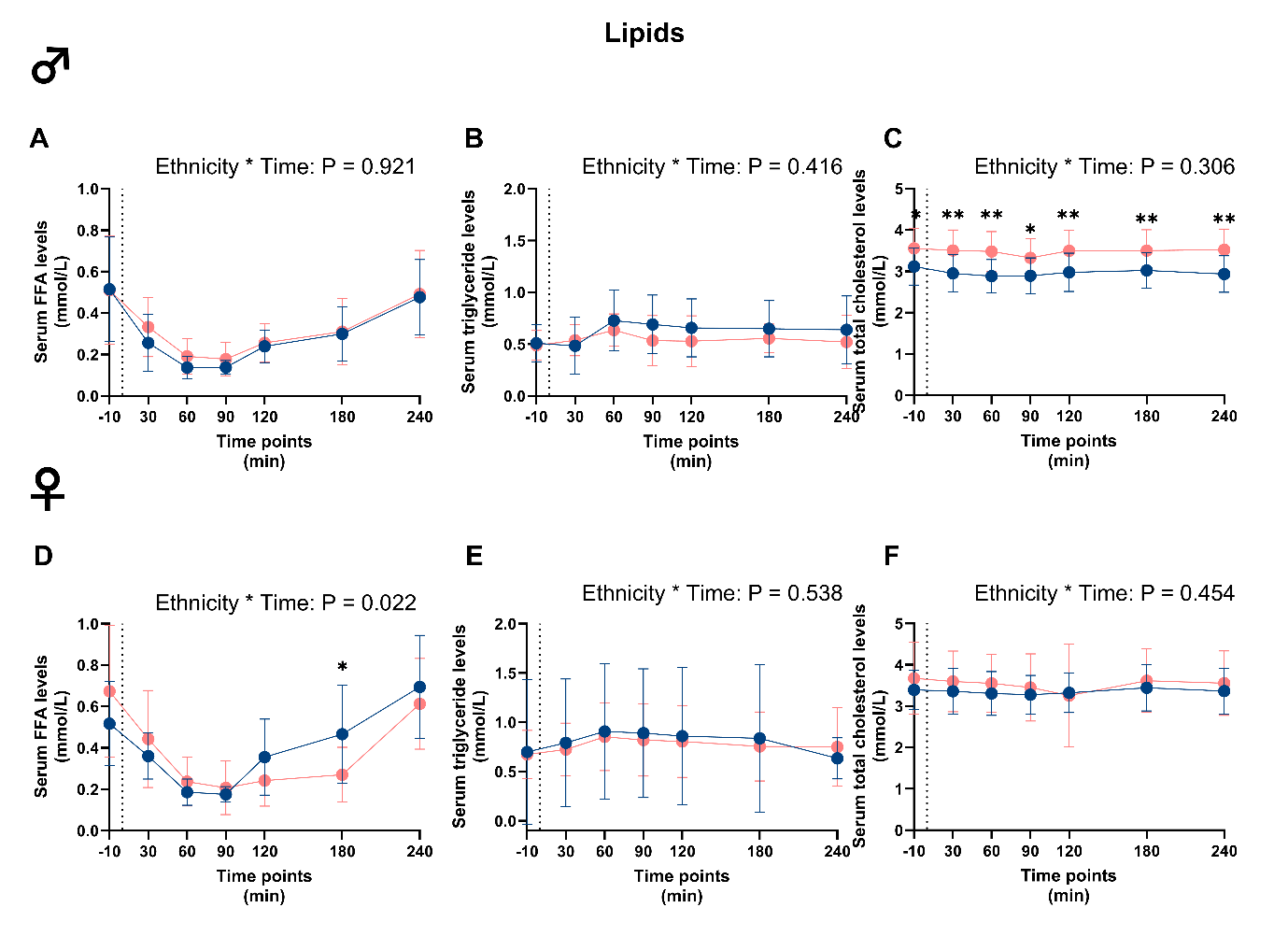


**Supplemental Figure 6. Free fatty acids, triglycerides, and total cholesterol excursions before and during a mixed meal tolerance test in South Asian and Europid males and females**

Line graphs showing in South Asian (n=12) compared to Europid (n=13) males the free fatty acid (FFA) (**A)**, triglyceride **(B)**, and total cholesterol **(C)** excursions before and during a mixed meal tolerance test (MMTT). Similarly, showing in South Asian (n=12) compared to Europid (n=12) females line graphs showing the FFA, triglyceride, and total cholesterol excursions during an MMTT. Circles represent means and deviations are the standard deviations. Blue circles, lines, and boxes represent Europids, and pink circles, lines, and boxes represent South Asians. The dotted line is the time of the ingestion of the liquid meal. We were unable to retrieve a blood sample of one South Asian female at two time points, and from one South Asian male, Europid male, one South Asian female, and one Europid female at one time point.
